# Factors associated with non-adherence to medications in systemic lupus erythematosus: Results from a Swedish survey

**DOI:** 10.1177/09612033241242692

**Published:** 2024-03-28

**Authors:** Sharzad Emamikia, Alvaro Gomez, Theodor Ådahl, Gunilla von Perner, Yvonne Enman, Katerina Chatzidionysiou, Elizabeth V. Arkema, Ioannis Parodis

**Affiliations:** 1 Division of Rheumatology, Department of Medicine Solna, 27106 Karolinska Institutet and Karolinska University Hospital, Stockholm, Sweden; 2 Department of Rheumatology, Faculty of Medicine and Health, 6233 Örebro University, Örebro, Sweden; 3 Clinical Epidemiology Division, Department of Medicine Solna, 27106 Karolinska Institutet, Stockholm, Sweden

**Keywords:** Systemic lupus erythematosus, adherence, compliance, treatment refusal, medication

## Abstract

**Objective:**

To identify determinants of medication non-adherence in a Swedish population of systemic lupus erythematosus (SLE).

**Methods:**

Patients with SLE from Karolinska and Örebro University Hospitals participated in a survey-based cross-sectional study. Demographics, disease activity, organ damage, HRQoL (LupusQol, EQ-5D-5 L), medication non-adherence (<80% on CQR-19 or MASRI) and beliefs about medicines (BMQ) were registered. MASRI was used to report adherence to different drugs/drug classes, categorised into (i) antimalarial agents (AMA), (ii) glucocorticoids and (iii) other SLE medications. Multivariable logistic regression adjusted for age, sex, disease activity and organ damage.

**Results:**

Among 205 respondents, the median age was 52.0 years (IQR: 34.0–70.0), 86.3% were women, 66.8% were non-adherent to their medications according to CQR-19, and 6.6% and 6.3% were non-adherent to AMA and glucocorticoids, respectively, according to MASRI. Positive beliefs about glucocorticoids (OR; 95% CI: 0.77; 0.59–0.99; *p* = .039) and medications overall (0.71; 0.52–0.97; *p* = .029) were protective against non-adherence to glucocorticoids. Anxiety/depression (3.09; 1.12–8.54; *p* = .029), medication concerns (1.12; 1.05–1.20; *p* < .001) and belief that medications are overused (1.30; 1.15–1.46; *p* < .001) or harmful (1.36; 1.19–1.56; *p* < .001) were associated with medication non-adherence (CQR-19); beliefs in the necessity of medications (0.73; 0.65–0.82; *p* < .001) and positive beliefs in medications were protective (0.72; 0.60–0.86; *p* < .001). No associations were found between other investigated factors and medication non-adherence.

**Conclusions:**

Beliefs about medications were a major determinant of medication non-adherence. Patient education may help alleviate the negative impact of misinformation/unawareness on adherence.

## Lay summary

Medication non-adherence is common among patients with systemic lupus erythematosus (SLE) worldwide. However, there is no universally accepted way of assessing and managing medication non-adherence. Also, little is known about factors contributing to non-adherence in patients with SLE. This study involved patients with SLE from two Swedish tertiary referral centres. We found that impaired health-related quality of life (HRQoL) captured by an instrument called LupusQoL negatively impacted adherence to antimalarial agents, glucocorticoids and other medications for SLE. Self-reported anxiety/depression using an instrument called EQ-5D-5L was associated with non-adherence to anti-rheumatic medications. Beliefs about medications captured by the BMQ had a strong impact on SLE patients’ adherence to anti-rheumatic medications. Our findings imply that patients with certain beliefs about medications could benefit from proper information about the necessity of medications and benefits from their usage, as a part of patient education. This may help alleviate the negative impact of misinformation or non-awareness on adherence to medications. In addition, patients with poor HRQoL should receive more attention in relation to medication adherence.

## Introduction

Adherence to medications can be challenging for people living with chronic diseases, and how to address non-adherence is ambiguous.^1^ Systemic lupus erythematosus (SLE) is a chronic disease that characteristically manifests in multiple organ systems, including the brain, kidneys, joints, skin, heart, blood and lungs.^2^ Several studies have reported proportions of patients with SLE who are non-adherent to medications; those range widely from 3% to 76% depending on the methods of assessment.^3,4^

Multiple treatments are used for managing SLE, based on organ affliction and degree of activity.^5,6^ Pharmacotherapeutic options include broad immunosuppressants and immunomodulatory agents.^7^ Long-term treatment with antimalarial agents (AMA), in particular hydroxychloroquine (HCQ), is recommended for all patients with SLE unless contraindicated for the prevention of flares and cardiovascular comorbidities,^8–12^ as well as for their documented effects in articular^13^ and cutaneous^14^ manifestations. Importantly, AMA are considered safe for use during pregnancy.^15^ Glucocorticoids (GCs) are used at varying doses; low doses are mainly used for disease control and symptom relief, while high doses are used to treat flares.^15^ Biological agents have been used during the last two decades.^16,17^ While therapeutic advancements pave the way towards a better prognosis and health-related quality of life (HRQoL) in people with SLE, non-adherence to medications remains a challenge.^3^

Non-adherence may be due to intentional or unintentional causes,^3^ with the latter often being related to forgetting to take the medications.^18^ Reasons for intentional non-adherence to medications include experience of side-effects,^19,20^ weight gain and hair loss have been found to most likely cause depression, which in turn can lead to medication non-adhrence.^21^ Other reasons include inability to pay for the medications,^20^ or disagreement with the treating physician regarding the need for pharmacological treatment, the latter commonly due to an untrusting relationship between patient and physician.^22^

Patients’ negative beliefs about medications as a whole or about particular medications are likely to be associated with non-adherence.^23^ Other factors that potentially have a negative impact in patients with SLE in particular include the fear of side-effects,^20,24,25^ concerns about medications that the patient is taking and beliefs that medications are generally harmful.^25,26^ Moreover, perceived inefficacy of medications is a common reason for discontinuing AMA treatment by patient initiative.^20,24,27^

Certain methods of assessing medication adherence are based on patient self-responses to questionnaires,^28–31^ for example, the generic questionnaires Morisky Medication Adherence Scale (MMAS),^28^ Medication Adherence Self-Report Inventory (MASRI),^31^ and the specific for rheumatic diseases Compliance Questionnaire of Rheumatology (CQR-19) questionnaire.^29,30^ Today, there is no universally accepted way of assessing medication non-adherence, but the electronic medication event monitoring (eMEM) system^32,33^ is oftentimes considered the gold standard for measuring medication adherence in research studies by means of a microchip that is placed in the cap of a medicine bottle, which records the date and time whenever the bottle was opened. Other methods used to determine adherence include drug availability estimates, for example, refill compliance based on pharmacy records aiding the calculation of the medication possession ratio (MPR) or proportion of days covered (PDC).^34,35^ Specifically for patients with SLE, measuring blood levels of HCQ is considered a reliable objective method.^36^

Importantly, inadequate adherence to medications may potentially lead to poor patient outcomes,^4,18,35,37–39^ for example, a higher risk for emergency hospital visits or hospitalisations,^18,35,37^ as well as poor long-term renal outcomes in patients with renal involvement.^38,39^ This imposes to the scientific community an urgent need to explore and in-depth understand the reasons underlying medication non-adherence within and across different SLE patient populations and take action to alleviate this phenomenon. In the present study, our aim was to determine factors that are associated with medication non-adherence in a Swedish SLE patient population, to subsequently suggest strategies for improving the level of adherence. Since specific measures of adherence to medications are not universally accepted, we also performed a correlation analysis between the MASRI and CQR-19 scales to investigate their interrelationship as a secondary objective.

## Methods

### Study setting and design

We designed a cross-sectional study, which was conducted between June 2021 and December 2021. The survey was advertised at the clinics. For the purpose of the study, a survey was distributed to the home addresses of adult patients (≥18 years of age) with SLE. The survey was developed together with two patient research partners residing in the survey distribution area and with Swedish as their mother language, including design and content adaptation (G.v.*P. and* Y.E.), and it was pretested on multiple occasions for content coherence and duration of completion by one (Y.E.). All information provided was self-reported by the study participants digitally or on paper, except for disease duration which was extracted from medical records. The data entry for the non-web-based surveys was done with caution and for the web-based surveys; the civic registration numbers were checked to prevent the same patient to participate more than once.

Data were collected and managed using the Research Electronic Data Capture (REDCap) system, where pseudonymised patient data were hosted on a password protected server at Karolinska Institutet.

### Selection of patients

Patients currently followed at the Karolinska University Hospital (*n* = 979) and Örebro University Hospital (*n* = 138) in Sweden were asked to participate in the study through a letter. The patients were identified through the International Classification of Diseases (ICD) coding system; patients with at least one ICD code indicating a diagnosis of SLE (M32.9 and M32.1) were deemed eligible to receive the letter inviting to participate in the study.

### Data collection and conceptual model

We collected information on demographics (age, sex, country of birth/origin, living situation, employment, education level, body mass index (BMI) and smoking status), medications (ongoing medication, prednisolone dose and number of medications per day), comorbidities, reasons for difficulties taking medications as prescribed, medication adherence, disease activity, organ damage, HRQoL and beliefs about medications.

**Figure 1.**
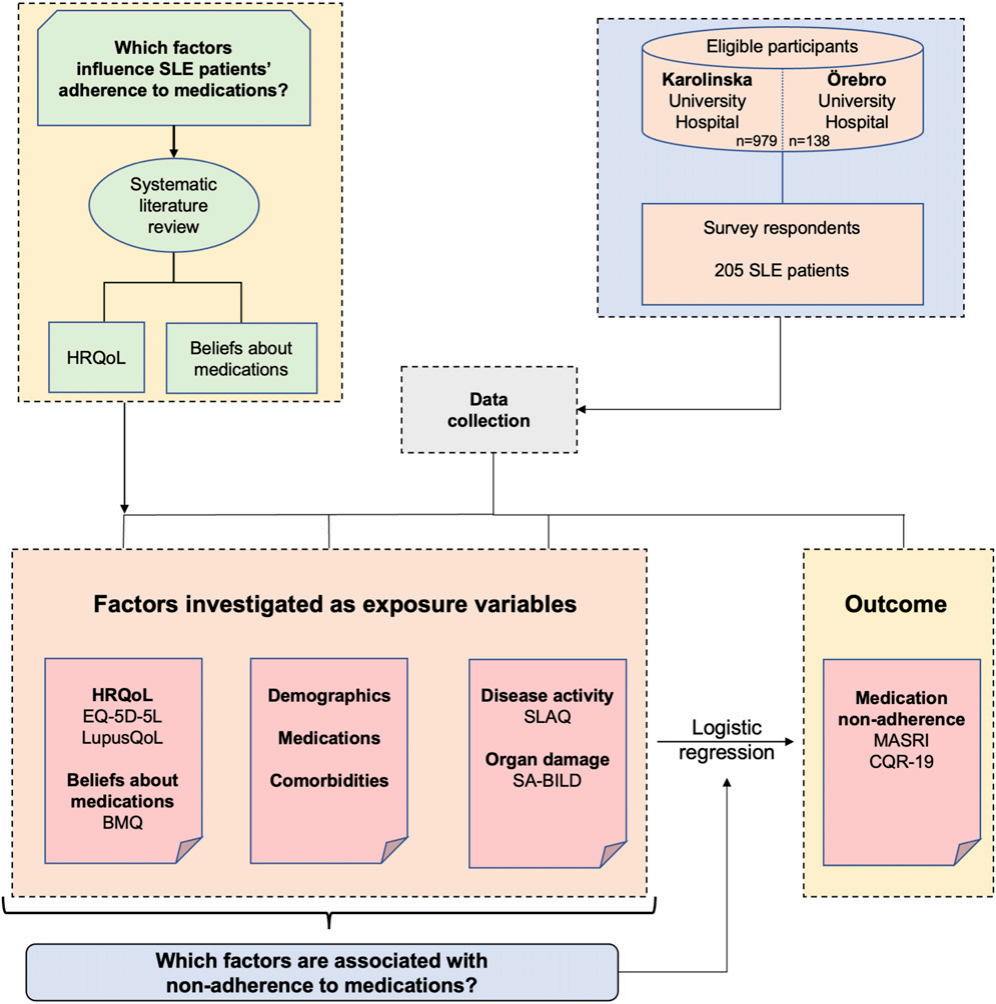
Study workflow. The diagram illustrates the study design and workflow, including a conceptual model for the selection of exposure variables in multivariable logistic regression models. HRQoL: Health-Related Quality of Life; BMQ: Beliefs about Medications Questionnaire; SLAQ; Systemic Lupus Assessment Questionnaire; SA-BILD: Self-Administered Brief Index of Damage; MASRI: medication adherence self-report inventory; CQR: Compliance Questionnaire Rheumatology.

Figure 1 illustrates a conceptual model for the selection of exposure variables in multivariable logistic regression models. In brief, based on previous literature, HRQoL and beliefs about medications were hypothesised to impact medication adherence and were therefore selected as variables to be investigated and covariates to be included in our multivariable regression models. Beyond those, several additional factors were investigated as exposure variables.

### Study outcome: Medication adherence

Medication adherence was assessed using the MASRI, a tool that was originally formed for patients affected by human immunodeficiency viruses (HIV) but has also been validated for patients with SLE.^40^ MASRI was used to report adherence separately to different drugs/drug classes, categorised into (i) antimalarial agents (AMA), (ii) glucocorticoids and (iii) SLE medications other than AMA or glucocorticoids. A detailed description of the MASRI is found in the Supplemental Material S1.

The CQR-19^29,30^ was also used to assess overall adherence to anti-rheumatic medications in general (including glucocorticoids, antimalarial agents, immunosuppressants and biologics), in order to account for the context of rheumatic disease. The CQR-19 was filled in once per patient and was intended to capture adherence to all anti-rheumatic medications. A description of the CQR-19 is provided in the Supplemental Material S2.

Within both MASRI and CQR-19, an adherence estimate ranging from 0 to 100 is calculated to reflect overall adherence. MASRI and CQR-19 levels below 80% denoted non-adherence to medications.^41^

### Factors investigated as exposure variables

#### Reasons for medication non-adherence

Reasons for medication non-adherence were collected in the survey with predefined response options. The items in the survey and the response options were determined based on a systematic literature review conducted in preparation of the present study. Importantly, while response options were predetermined, patients were also given the alternative ‘other reasons’ where they could fill in reasons other than those predefined, in the form of free text.

#### Disease activity

Disease activity was measured using the Systemic Lupus Activity Questionnaire (SLAQ),^42,43^ a patient-reported measure that has been validated for SLE patients and is based on the physician-administered Systemic Lupus Activity Measure (SLAM).^44^ A description of the SLAQ is provided in the Supplemental Material S3.

#### Flares

Flares within the preceding three months were self-reported using the SLAQ.

#### Organ damage

Organ damage was estimated using the 28-item Self-Administered Brief Index of Lupus Damage (SA-BILD), which is validated for use in SLE.^45^ The SA-BILD was developed as a self-reported proxy for the Systemic Lupus International Collaborating Clinics (SLICC)/American College of Rheumatology (ACR) Damage Index (SDI).^46^ The initial version of BILD^47,48^ was designed to be used for obtaining information from patients over the telephone or in person, and the self-administered version was developed for use in surveys.^45^

#### Health-related quality of life

For the assessment of HRQoL, the survey included the five-level response scale version of the generic EuroQoL-5D (EQ-5D-5L),^49^ which consists of a descriptive part and VAS. The descriptive part comprises five health dimensions, that is, mobility, self-care, usual activities, pain/discomfort and anxiety/depression, with patient responses being weighted to generate a utility index score from below 0 to 1, where 1 represents full health state. The VAS is scored from 0 to 100 (worst to best imaginable health).

In addition to EQ-5D-5L, the SLE-specific LupusQoL was used. The LupusQoL comprises 34 items across eight domains, that is, physical health (8 items), emotional health (6 items), body image (5 items), pain (3 items), planning of usual activities (3 items), fatigue (4 items), intimate relationships (2 items) and burden to others (3 items), yielding a score from 0 to 100 (worst to best imaginable health).^50^

#### Beliefs about medications

The Beliefs about Medicines Questionnaire (BMQ)^23,25,26,51–54^ comprises two compartments; one compartment covers specific medications that the patient is currently taking, with subscales capturing necessity and concern, and the other compartment covers views on medications in general, with subscales capturing harm, overuse and general beliefs. The statements are anchored on 5-point Likert scales with 1 denoting that the patient strongly disagrees, 2 being equivalent to disagreement, 3 denoting uncertainty, 4 denoting agreement with the statement and 5 denoting that the patient strongly agrees. Higher BMQ scores indicate stronger beliefs. The BMQ was filled in only once by each patient and was intended to capture beliefs about medications in general rather than specific drugs or drug classes.

### Translation of questionnaires

Swedish versions of the SA-BILD, MASRI, CQR-19 and the complete BMQ were neither available in the literature nor publicly available. Hence, we translated those into Swedish through the KI-trusted CBG Konsult & Information (Supplemental Material S4–S7). The translation method is described in Supplemental Material S8.

The custom survey questions in English and Swedish are presented in Supplemental Material S9.

### Statistical analyses

Descriptive statistics were expressed as medians and the corresponding interquartile range (IQR) or means and standard deviation (SD). Numbers and percentages were reported for categorical variables. We performed a complete case analysis for each separate model. The amount of missing data for each investigated variable is described in Table 1. Associations with binary non-adherence were estimated using logistic regression analysis where we followed a two-step process for adjustments. The initial models were univariable. Next, we employed models adjusting for age, sex, organ damage and disease activity. These factors had confounding potentiality based on previous research.^3,26,55,56^ Factors investigated in relation to medication non-adherence included country of birth, living situation, employment status, education level, BMI, smoking status, disease duration, HRQoL and beliefs about medications. The Spearman’s rank correlation coefficient was used to assess the correlation between MASRI and CQR-19 scores. *p* values below 0.05 were deemed statistically significant. To adjust for multiple comparisons, a Bonferroni correction was applied yielding levels of significance of *p* values below 0.002 (critical *p* value: 0.05; number of tests: 27). The statistical analyses were performed using IBM SPSS Statistics 28 software (IBM Corp., Armonk, New York, USA).

**Table 1.** Sociodemographic and disease characteristics of survey respondents.

Study population (*N* = 205)	
Median age, years (IQR)	52.0 (34.5–70.5)
Female sex, *n* (%)	177 (86.3)
Median disease duration, years (IQR)	12 (5–22)
Household, *n* (%)	
Single	58 (28.3)
Country of birth, *n* (%)	
Sweden	164 (80.4)
Missing	1 (0.5)
Employment status, *n* (%)	
Employed	118 (57.6)
Unemployed^a^	87 (42.4)
Student	5 (6.0)
Retired	69 (83.1)
Other reason	13 (15.7)
Education level, *n* (%)	
University	126 (62.7)
High school	57 (28.4)
Less than high school diploma	18 (9.0)
Missing	4 (2.0)
Smoking status, *n* (%)	
Current smokers	9 (4.4)
Former smokers	82 (40.0)
Never smokers	114 (55.6)
Comorbidities, *n* (%)	
Hypertension	58 (28.3)
Sjögren’s syndrome	49 (23.9)
Osteoarthritis	36 (17.6)
Osteopenia or osteoporosis	20 (9.8)
Fibromyalgia	18 (8.8)
Depression	13 (6.3)
Hyperlipidaemia	13 (6.3)
Rheumatoid arthritis	10 (4.9)
Diabetes	9 (4.4)
Number of medications, *n* (%)	
4–5	35 (17.2)
>5	94 (46.1)
Missing	1 (0.5)
Medications, *n* (%)	
Glucocorticoids	113 (55.1)
Prednisolone	111 (54.1)
Median prednisolone equivalent dose (mg/day)	2.5 (0.0–5.0)
Antimalarial agents	154 (75.1)
HCQ	150 (73.2)
Immunosuppressive or biological agents	75 (36.6)
Azathioprine	23 (11.2)
Mycophenolate mofetil	23 (11.2)
Methotrexate	18 (8.8)
Belimumab	9 (4.4)
Rituximab	11 (5.4)

Data are presented as numbers (%) or the median (IQR). Note that one survey respondent may contribute to several sub-categories.

**IQR:** interquartile range; **HCQ:** hydroxychloroquine; **AMA:** antimalarial agents; **GCs:** glucocorticoids.

^a^The categories under unemployed are student, retired and other reasons.

### Ethics

The study design and conduct complied with the ethical principles of the Declaration of Helsinki. The study protocol was approved by the Swedish Ethical Review Authority on April 1st, 2021 (reference number: 2021-00662). Informed consent was obtained from all study participants prior to enrolment.

### Patient involvement

Patient research partners (Y.E. and G.v.P.) were involved in the research process, including design, study conduct, interpretation of results and dissemination.

## Results

### Study population

Of 1177 patients with SLE who were deemed eligible for participation and received our invitation to participate, a total of 205 patients (17.4%) responded to the survey. Unique respondents were determined by civic registration numbers and informed consents. The survey respondents’ median age was 52.0 years (IQR: 34.0–70.0), they were predominantly females (86.3%).

The patients had a median SLE duration of 12.0 years (IQR: 5.0–22.0). Most of the study participants were born in Sweden (80.4%), were living together with someone (71.7%), were non-smokers (95.6%), had a university-level education (62.7%) and were currently employed (57.6%). Among the unemployed, a substantial proportion of patients were retired (83.1%). Comorbidities existed in 80.0% of the study population, with hypertension being the most common comorbidity (28.3%). A total of 78 patients (38.0%) were overweight (defined according to WHO as a BMI ≥25 kg/m^2^). Details about the demographics of the cohort are shown in Table 1.

### Medications

As reported by the patients, AMA were used by 154 patients (75.1%) and GCs by 113 (55.1%). A total of 75 patients (36.6%) were on treatment with synthetic immunosuppressants, which comprised azathioprine, mycophenolate mofetil, and methotrexate, or biological agents (belimumab or rituximab; Table 1). Seventy-eight patients (38.0%) were on both AMA and GCs.

### Medication adherence

Inadequate levels of adherence to AMA intake according to the VAS scale of MASRI (<80%) was reported by 10/151 (6.6%) patients (median adherence: 100 [IQR: 95–100]); 91/151 (60.3%) reported 100% adherence to AMA. The proportion of patients on GCs (*n* = 111) who reported inadequate (<80%) levels of adherence to GCs was 7/111 (6.3%) (median adherence: 100 [IQR: 99–100]); 81/111 (73.0%) reported 100% adherence. Concerning medications prescribed for SLE other than AMA or GCs, 13/128 (10.2%) reported inadequate levels of adherence (median adherence: 100 [IQR: 95–100]); 73/128 (57.0%) reported 100% adherence.

When measured with the CQR-19, 135/202 patients (66.8%) reported inadequate levels of adherence to their anti-rheumatic medications (<80%). The mean adherence level was 72.5 ± 13.2, with only one patient stating 100% adherence.

### Correlation between adherence levels by MASRI and CQR-19

Adherence levels assessed by CQR-19 showed a moderate correlation with those assessed using MASRI for AMA (ρ = 0.47; *p* < .001), GCs (ρ = 0.34; *p* < .001) and SLE-related medications other than AMA or GCs (ρ = 0.41; *p* < .001).

### Reasons for medication non-adherence

The most common reason for not taking medications as prescribed was ‘being worried about potential side-effects’, which was reported by 43/205 (21.0%) of study participants. The second most common reason for non-adherence was ‘too many different medications to keep track of’, reported by 11/205 of the patients (5.4%). Results are detailed in Table 2.

**Table 2.** Reasons for missing medications as reported by survey respondents.

Reasons for missing medications	Study population (*N* = 205)
Worried about side-effects	43 (21.0)
Too many different medications to keep track on	11 (5.4)
When feeling unwell	10 (4.9)
Unhappy with physician/healthcare	9 (4.4)
Too many different timeslots to keep track on	9 (4.4)
Inactive disease	9 (4.4)
I Believe my symptoms will not be improved	8 (3.9)
Worried to increase the risk of a more severe Covid-19 infection	7 (3.4)
Afraid to increase the risk of developing Covid-19 infection	4 (2.0)
It takes too long to for my symptoms to be improved	3 (1.5)
Financial issues	1 (0.5)
Other reasons	19 (9.3)
I Forget	9 (47.4)
I take medications only when flares remind me	1 (5.3)
I experience actual side-effects	7 (36.8)
I don’t feel that I need them	2 (10.5)
Reason not stated	0 (0.0)

Data are presented as numbers (%). Covid-19: Coronavirus Disease 2019.

### Disease activity and organ damage

About half of the patients (110/200; 55%) reported that they had not experienced flares during the preceding three months. Among patients who had experienced flares, mild flares were the most common ones, experienced by 27.5% (55/200), followed by moderate flares (29/200; 14.5%). Severe flares had occurred in 3.0% of the study population (6/200).

Details on the SLE disease activity and organ damage are presented in Supplemental Table S1.

### Health-related quality of life

The EQ-5D-5 L utility index and VAS scores are presented in Supplemental Table S1. The lowest LupusQoL median score was 75 for fatigue and the highest was 100 for intimate relationships (Supplemental Table S1).

### Beliefs about medicines

In our study population, 113/198 study participants (57.0%) either strongly agreed or agreed that it is necessary to take medications to treat SLE and that there is a personal need of the medication to maintain good health as assessed with the BMQ. In total, 36/198 patients (18.2%) fully agreed with the necessity of medications. The subscale ‘beliefs in the necessity of specific medications’ yielded a mean score of 20.0 ± 4.0 (max: 25). Overall, 15/198 patients (7.6%) either strongly agreed or agreed with concerns about potential negative effects of the medications but only 2/198 patients (1.0%) strongly agreed with the statement, with a total mean score of 15.5 ± 5.5 (max: 30).

Ten of 200 patients (5.0%) either strongly agreed or agreed with statements that medications are generally overused, implying negative views on how medications are used in the clinical setting. The subscale ‘beliefs that medications are generally overused’ yielded a mean score of 10.6 ± 3.1 (max: 20). Of 200 patients, 38 (19.0%) agreed on the view of medicines as fundamentally harmful, with 7/200 of the patients (3.5%) reporting that they strongly agree with this statement. Patient reports on this subscale yielded a mean score of 8.7 ± 3.0 (max: 20).

A generally positive belief in medications was reported by 132/200 of the patients (66.0%), of which 11/200 (5.5%) strongly believed in medications, yielding a mean score of the subscale of ‘general positive belief in medications’ 16.5 ± 2.1 (max: 20).

### Factors associated with non-adherence

**Figure 2.**
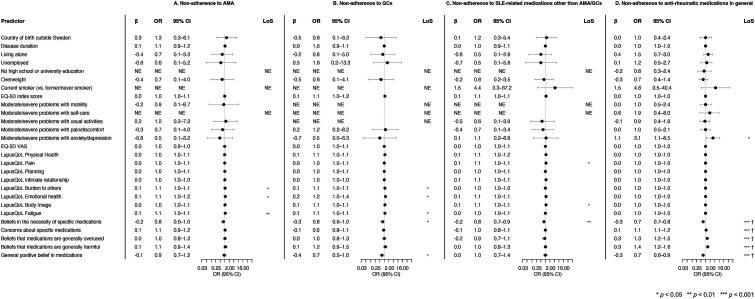
Associations with non-adherence to medications in fully adjusted models. Odds ratios and 95% confidence intervals from logistic regression analyses adjusted for age, sex, disease activity and organ damage, investigating associations with non-adherence to antimalarial agents (a), glucocorticoids (b), SLE-related medications other than antimalarial agents or glucocorticoids (c), all the above as assessed with MASRI, as well as anti-rheumatic medications in general as assessed with CQR-19 (d). Each row represents a separate adjusted model. Asterisks indicate statistically significant associations and crosses indicate associations that remained significant after Bonferroni correction. A higher OR indicates higher risk of medication non-adherence. Overweight was defined as a body mass index of ≥25 kg/m^2^. Beliefs about medications were assessed with the Beliefs about Medicines Questionnaire (BMQ). MASRI: medication adherence self-report inventory; OR: odds ratio; LoS: level of significance; VAS: visual analogue scale; NE: not estimable; CQR-19: compliance questionnaire rheumatology.

Next, factors potentially associated with non-adherence were investigated using logistic regression analysis. Univariable analyses and multivariable models for each one of the variables of interest are adjusted for age, sex, disease activity, and organ damage and are displayed in Supplemental Tables S2–S5. Results are also illustrated in Figure 2.

In multivariable logistic regression models including age, sex, disease activity estimated by SLAQ scores and organ damage estimated by SA-BILD scores as covariates, the perception of being a ‘burden to others’ as assessed with the LupusQoL (OR: 1.05; 95% CI: 1.00–1.09), ‘emotional health’ aspects by LupusQoL (OR: 1.09; 95% CI: 1.02–1.18) and fatigue by LupusQoL (OR: 1.08; 95% CI: 1.02–1.14) were associated with non-adherence to AMA as defined by the MASRI (Figure 2(a); Supplemental Table S2). Similarly, perception of being a ‘burden to others’ as assessed using the LupusQoL was associated with non-adherence to GCs (OR: 1.05; 95% CI: 1.00–1.10), as were ‘emotional health’ aspects by LupusQoL (OR: 1.18; 95% CI: 1.03–1.36) and fatigue by LupusQoL (OR: 1.07; 95% CI: 1.01–1.13), whereas beliefs in the necessity of specific medications, that is, GCs (OR: 0.77; 95% CI: 0.59–0.99) and general positive beliefs about medications (OR: 0.71; 95% CI: 0.52–0.97) were protective against non-adherence to GCs (Figure 2(b); Supplemental Table S3). Beliefs in the necessity of specific medications were protective against non-adherence to medications other than AMA or GCs (OR: 0.79; 95% CI: 0.68–0.93; Figure 2(c); Supplemental Table S4), whereas higher LupusQoL pain scores (OR: 1.06; 95% CI: 1.00–1.12) and body image scores were associated with non-adherence to medications other than AMA or GCs (OR: 1.07; 95% CI: 1.00–1.14). Lastly, in these models, all aspects concerning beliefs about medications in general or beliefs about particular medicines remained significantly associated with anti-rheumatic medication non-adherence as defined by the CQR-19, as did experience of anxiety or depression according to the EQ-5D (OR: 3.09; 95% CI: 1.12–8.54; Figure 2(d); Supplemental Table S5). Beliefs about medications in association with non-adherence assessed with the CQR-19 remained significant after Bonferroni correction.

## Discussion

In this study, we reported on the extent of non-adherence to medications in patients with SLE from two Swedish tertiary referral centres, and investigated the impact of different factors on medication non-adherence. The extent of non-adherence differed considerably between MASRI and CQR-19. In this study population, beliefs about medications constituted a major determinant of adherence to anti-rheumatic medications. In addition to this, impaired HRQoL had a negative impact on medication adherence.

Based on the findings of this work, lack of information or misinformation about the drugs and the disease may contribute to poor adherence to prescribed medications, in turn resulting in low drug efficacy and worsened disease activity as well as worsened HRQoL. Subsequently, poor HRQoL appears to be an important determinant of non-adherence to medications, perpetuating a cause and effect vicious circle.

Proportions of patients adherent to their anti-rheumatic medications as assessed with the CQR-19 (33.2%) were substantially lower compared with those yielded using the MASRI, which exceeded 80% across different medications. Moreover, adherence levels based on MASRI and CQR-19 were not strongly correlated. This discrepancy between the two indices may indicate that MASRI and CQR-19 capture different aspects of medication non-adherence, and that the cut-off determining non-adherence by CQR-19 may be stringent for populations of SLE. In this regard, it is important to interpret the results with caution, keeping in mind that adherence levels by MASRI rely on the respondent’s spontaneous self-perception of adherence, whereas adherence levels by the CQR-19 are based on the respondent’s level of agreement with specific statements some of which are related to beliefs. Thus, the CQR-19 is a complex questionnaire, likely yielding a less conscious impact on the final score from the respondent. Also, the strong associations regarding beliefs about medications and non-adherence assessed with CQR-19 could be related to the higher proportion of non-adherent patients compared to non-adherence assessed with the MASRI.

Similar to our study, other studies on adherence to medications using the MASRI documented high proportions of patients with SLE reporting adequate levels of adherence to HCQ in an international cohort (76.6%),^57^ to AMA in another study from Canada (76.2%) where the adherence levels were also confirmed with pill counts in a proportion of the patients,^58^ and to HCQ in a study from France (86.2%).^59^ The same study from Canada showed that 46.4% of the patients were adequately adherent to GCs,^58^ in contrast with our study (93.7%).

Proportions of patients with SLE reporting adequate levels of adherence according to CQR-19 were overall higher in previous studies compared with our results; two studies from the United States (US) reported nearly 50% of SLE patients to be adequately adherent, one of those also comprising patients with rheumatoid arthritis (RA).^19,60^ Two studies from China reported adequate adherence in 43% and 48% of patients with SLE.^61,62^ In the validation study of CQR-19 that assessed medication adherence in patients with rheumatic diseases other than SLE,^30^ 52% of the overall population reported adequate levels of adherence. The differences in proportions of adherent patients between our study and other investigations may be due to various reasons, including discrepancies in cultural and socioeconomic facets across SLE populations, which are likely to impact on actual adherence and perception of adherence. One sociodemographic factor that has an impact on adherence to medications is patient’s education; lower levels of education have been associated with non-adherence, but some studies have shown that higher education levels may also be associated with non-adherence.^19,56,60,61,63,64^ In our study, we found no association between the participants’ level of education and medication non-adherence. One of the possible explanations for this discrepancy may be the limited sample size. Another explanation may be the overall higher proportion of study participants with university education in our cohort, compared, for instance, with SLE populations in previous investigations from Sweden.^64,65^ In fact, higher educated people have been shown to be more inclined to question the physician’s reasoning and prescription, or to adjust the dose of a medication by their own initiative.^64^

Our results indicate that different aspects of beliefs about medications constitute factors with a major impact on SLE patients’ adherence to medications. Better information to patients could potentially be delivered through patient organisations. Overall, believing in the necessity of specific medications and having a general positive belief in medications yielded a favourable association with medication adherence, whereas having concerns about specific medications or believing that medications are generally overused and harmful yielded a negative association. Importantly, the most common reason for non-adherence was concerns about side-effects, which is expected to impact patients’ beliefs about medications. To this end, it is important to underscore the multilateral interrelationship across factors identified to associate with medication non-adherence. A 3-month randomised controlled trial involving patients with rheumatoid arthritis that investigated how patients’ attitudes towards disease-modifying anti-rheumatic drugs (DMARDs) alter by using an electronic game showed no impact of the game on patient’s attitudes (^66^). It would be intriguing to enrich the future research agenda in the field of SLE with prospective investigations of the effect of altering patients’ beliefs about medications on their adherence, as well as investigations of means by which patients’ beliefs about medications may alter over time.

Our findings suggest that adherence to AMA is not associated with demographics, disease activity, organ damage or beliefs about medications. In contrast, adherence to GCs was demonstrated to be enhanced by beliefs in the necessity of medications, as well as by an overall positive view in medications. This finding indicates that beliefs in different medications can vary and perhaps have an impact on medication adherence. For instance, different expected efficacy or different potential side-effects may differentially impact patients’ attitude towards medications. Another study that investigated adherence to medications using the MASRI made a distinction between intentional and unintentional non-adherence; in that study, patients who were intentionally non-adherent also had stronger beliefs about medication overuse compared with patients who were not.^25^ The intentionally non-adherent patients were also more concerned about possible side-effects of medications compared with the unintentionally non-adherent patients.^25^ Compared with a study of patients with SLE conducted in China,^53^ the patients in our study had overall stronger beliefs in the necessity of medications and fewer concerns about their medications. Regarding the use of HCQ in particular, a poor belief in the necessity of HCQ has been shown to be associated with non-adherence,^54^ which we were unable to corroborate in the present study. Beliefs about harmfulness of medications in general terms were also shown to have a negative impact on medication adherence in a previous study of patients with SLE from Germany.^26^

Experience of fatigue as captured by the SLE-specific LupusQoL was found to be associated with non-adherence to AMA and GCs. Moreover, it is worth mentioning that other studies have identified depression as one of the causes of poor adherence to medications in patients with SLE,^67,68^ which was confirmed in the present investigation. Collectively, interventions to enhance adherence to medications emerge as a means towards a better HRQoL experience for patients with SLE. This aligns with treat-to-target concepts in SLE,^69^ as attainment of remission and low disease activity were recently shown to contribute to better HRQoL outcomes.^70^

### Limitations and strengths

One of the limitations of this study was its cross-sectional nature; hence, no causal relationships could be established. Nevertheless, these descriptive data on the extent of medication non-adherence in Swedish SLE populations are highly informative. Moreover, survey research is potentially limited by selection bias; patients who consider themselves adherent may be more prone to respond. Most participants had a high median age and were born in Sweden. Age has been shown to be important from two distinct perspectives; not only younger patients^26,59^ but also patients who have been diagnosed at a young age^57^ are more prone to be non-adherent. Furthermore, people with a shorter SLE disease duration have also been shown to be more inclined to non-adherence.^18^

Furthermore, the response rate to the survey was relatively low and the majority of survey respondents had a high educational level, which both limit the external validity of the findings. Moreover, the majority of study participants were of European origin, and all resided in mid Sweden (Stockholm or Örebro), which made investigations of commonalities and differences across ancestries or cultures impossible with this material. Hence, interpretation of the results in the context of their applicability to SLE populations across the globe should be made with caution.

The level of non-adherence was arbitrarily set to below 80% for both CQR-19 and MASRI; however, this choice of threshold was made in order to be consistent with multiple previous studies of SLE,^40,56–58,71,72^ which facilitates comparisons of findings across study populations. It is important to mention that the rather low proportions of non-adherent patients according to MASRI could partly be due to patients who were prescribed a certain medication but did not take that medication at all. These patients may have skipped the MASRI question for that specific medication, likely leading to an overall underestimation of non-adherence. Moreover, in addition to non-adherence as a discrete state, we also analysed adherence levels on continuous scales. Translating questionnaires from their original language to a new one, in this case to Swedish, may have some disadvantages; for instance, the translated versions of the BMQ to Scandinavian languages (including Swedish) have been questioned^51^ as some items in the questionnaire do not translate sufficiently well and are language-specific. Thus, an important setback was that some instruments that were translated for the purpose of this study were not validated in Swedish context. However, it is important to keep in mind that questionnaires have limitations even in their original versions. Importantly, we used rigorous translation methodology, and the use of some instruments in a Swedish SLE population for the first time provides some novelty.

Importantly, we assessed adherence using questionnaires since measuring medication adherence with drug concentrations or eMEM was not feasible in our study; eMEM requires a specific apparatus, which we chose to not use in order to avoid interference with the participants’ typical routines while taking their medications. However, the MASRI has been shown to correlate with pharmacy refill adherence rates,^40^ but the total CQR-19 score (0–100) has not been shown to correlate with adherence measured with eMEM.^30^ Both generic and disease-specific questionnaires were used in our survey, contributing to a collection of granular and multifaceted data. Importantly, the MASRI and the CQR-19 are not interchangeable as the construction of the questionnaires is different. While MASRI can be used to capture adherence to specific medications, CQR-19 is designed to capture overall adherence to medications in general in the context of rheumatic disease.

Also, the sex and age distributions of the survey respondents were comparable with those of the Karolinska SLE cohort^73^ as well as prevalent SLE cases from the National Patient Register, where 86.8% of SLE patients were women and the median age was 56.7 (IQR: 43.4–68.2) based on 6598 individuals with at least two visits with an ICD code for SLE as of December 2022 (personal communication: E.A.).

### Clinical relevance

Medication non-adherence can potentially be harmful for the patients. For instance, poor adherence to medications may lead to poor disease control, resulting in turn to increased morbidity, or may be mistaken by physicians for lack of response to medications, potentially leading to unnecessarily increased doses in prescriptions.^18,37–39,74^ Today, the patients’ adherence to medications is examined sporadically in real-life clinical settings, and in most instances based on the treating physician’s perception. The BMQ tool may prove to be useful for screening and detecting a risk for non-adherence, as also suggested for patients with RA.^75^ In addition to the above, clinicians could more effectively discuss potential side-effects of medications with SLE patients to ensure that patients are well-informed. This would also limit the patients’ need of seeking information about the medications from less reliable sources.

## Conclusions

In this predominantly middle-aged SLE patient population from Sweden with a long disease duration, relatively mild disease activity and high educational level, beliefs about medications were found to be a major determinant of adherence to anti-rheumatic medications. Hence, while validations are needed to confirm the findings in other SLE populations, our results imply that systematic efforts to educate patients and involve them in decision-making processes would be useful for alleviating the negative impact that misinformation or lack of awareness have on their adherence. Moreover, poor HRQoL experience was also found to contribute to medication non-adherence, suggesting that efforts for enhancing SLE patients’ experience of HRQoL as a part of SLE management may contribute to better disease outcomes through mitigation of non-adherence.

## Supplemental Material

Supplemental Material - Factors associated with non-adherence to medications in systemic lupus erythematosus: Results from a Swedish survey Supplemental Material for Factors associated with non-adherence to medications in systemic lupus erythematosus: Results from a Swedish survey by Sharzad Emamikia, Alvaro Gomez, Theodor Ådahl, Gunilla von Perner, Yvonne Enman, Katerina Chatzidionysiou, Elizabeth V. Arkema, Ioannis Parodis in Lupus.
