# Supplementary material for: Factors associated with non-adherence to medications in systemic lupus erythematosus: Results from a Swedish survey
Source: Lupus. 2024 Mar 28;33(6):615–28. doi: 10.1177/09612033241242692 (PMC11015713; doi:10.1177/09612033241242692)
Supplement: Supplemental Material - Factors associated with non-adherence to medications in systemic lupus erythematosus: Results from a Swedish survey [file sj-pdf-1-lup-10.1177_09612033241242692.pdf]

Factors associated with non-adherence to medications in systemic lupus erythematosus: results from a Swedish survey

SUPPLEMENTAL MATERIAL

Supplemental Material S1. Medication Adherence Self-Report Inventory (MASRI).

The MASRI is a 12- item questionnaire containing two parts of 6 items each: part A addresses frequency and part B correct timing of medication intake. Part A consists of specific questions on medication intake that help the patient estimate an adherence level to next accurately fill in a Visual Analogue Scale (VAS). The VAS ranges from 0 to 100 (minimum to maximum adherence level). Part B is focused on the exact timing of medication intake, which was less relevant in the context of treatment for SLE and was dropped in this study. The MASRI was filled in separately for antimalarials, glucocorticoids, and use of medications for SLE other than antimalarials or glucocorticoids.

Supplemental Material S2. Compliance Questionnaire Rheumatology-19 (CQR-19).

The CQR-19 includes nineteen statements on views on medications stated by patients with rheumatic disease which the patient can agree or disagree with, and has been validated against electronic medication event monitoring (eMEM) system (1). The CQR-19 is frequently used in studies of SLE (2-6).

Supplemental Material S3. Systemic Lupus Activity Questionnaire (SLAQ).

The SLAQ (7, 8) comprises a patient global assessment with one question about the presence and severity of a lupus flare over the past three months, and a symptom score based on 24 items corresponding to symptoms of SLE during the preceding three months. The symptom score ranges from 0 to 24 depending on the presence of the respective symptom, with low scores representing low disease activity. The SLAQ also comprises the SLAQ score, which is calculated taking into account the severity in each SLAQ item. The SLAQ score ranges from 0 to 47. Finally, SLAQ includes a numerical rating scale (0–10) where the patients rate the activity of their disease based on an overall perception of the disease over the past three months.

Supplemental Material S4. Self-Administered Brief Index of Lupus Damage (SA-BILD) in Swedish.

I denna undersökning samlar vi in information om symtom som du kan ha upplevt i relation till din lupus.
Oroa dig inte om det är några medicinska ord du inte förstår. Det innebär oftast att du inte har det problem som frågan berör.

**Ögon**	
Har en ögonläkare någon gång sagt att du har något fel på näthinnan i ögat på grund av din lupus? (Näthinnan sitter längst bak i ögat.)	◯ Ja<br>◯ Nej
Har en läkare någon gång sagt att du har starr i ögat?	◯ Ja<br>◯ Nej

**Hjärna**<br><br>**Har en läkare någon gång sagt att du har något av följande symtom?**	
En psykos?	◯ Ja<br>◯ Nej
Anfall?	◯ Ja<br>◯ Nej
Har du någon gång behövt ta medicin mot anfall i minst 6 månader?	◯ Ja<br>◯ Nej
Stroke?<br>(Detta omfattar inte TIA, det vill säga transitorisk ischemisk attack.)	◯ Ja<br>◯ Nej
Har du någon gång haft mer än 1 stroke med minst 6 månaders mellanrum?	◯ Ja<br>◯ Nej
Förlamning i armarna eller benen som var så allvarlig att du behövde ligga på sjukhus?<br>(Detta kallas även transversell myelit, och är ett sällsynt tillstånd som orsakas av inflammation i ryggmärgen.)	◯ Ja<br>◯ Nej
Orsakades denna förlamning av en stroke eller multipel skleros?	◯ Ja<br>◯ Nej

Factors associated with non-adherence to medications in systemic lupus erythematosus: results from a Swedish survey

**Njurar**	
Har du genomgått en njurtransplantation?	◯ Ja<br>◯ Nej
Har du fått dialys i 6 månader eller längre?	◯ Ja<br>◯ Nej

**Lungor**<br><br>**Har en läkare någon gång sagt att du har något av följande?**	
Pulmonell hypertension, vilket är högt blodtryck i lungorna?<br>(Detta skiljer sig från vanlig hypertension, det vill säga högt blodtryck. Diagnosen inleds med en EKG- eller ultraljudsundersökning av hjärtat, inte med en blodtrycksmanschett.)	◯ Ja<br>◯ Nej
Ett allvarligt lungtillstånd, till exempel fibros eller interstitiell lungsjukdom?<br>(Detta omfattar inte lunginflammation, astma, emfysem, lungsäcksinflammation, KOL eller bronkit.)	◯ Ja<br>◯ Nej

**Hjärta**	
Har du genomgått bypassoperation av kärl eller hjärta?	◯ Ja<br>◯ Nej
Har en läkare någon gång sagt att du har en hjärtsjukdom, inklusive kärlkramp eller hjärtsvikt?	◯ Ja<br>◯ Nej
Har en läkare någon gång sagt att du har fått en hjärtinfarkt?	◯ Ja<br>◯ Nej
Har du någon gång haft mer än 1 hjärtinfarkt med minst 6 månaders mellanrum?	◯ Ja<br>◯ Nej
Har en läkare någon gång sagt att du har drabbats av perikardit, vilket är en inflammation i säcken runt hjärtat, som varade i 6 månader eller längre?	◯ Ja<br>◯ Nej

**Blodkärl**<br><br>**Har du haft något av följande symtom på grund av din lupus?**	
Avmagring i fingertopparna?	◯ Ja<br>◯ Nej
Förlust av ett finger, en tå, eller en del av en arm eller ett ben som inte orsakades av en olycka?	◯ Ja<br>◯ Nej
Djup ventrombos (DVT), det vill säga blodpropp, i en arm eller ett ben?	◯ Ja<br>◯ Nej

**Mage och tarmar**<br><br>**Har du på grund av din lupus genomgått bukkirurgiskt ingrepp i:**	
Matstrupe	◯ Ja<br>◯ Nej
Mage	◯ Ja<br>◯ Nej
Tunntarm	◯ Ja<br>◯ Nej
Tjocktarm	◯ Ja<br>◯ Nej
Mjälte	◯ Ja<br>◯ Nej
Lever	◯ Ja<br>◯ Nej
Bukspottkörtel	◯ Ja<br>◯ Nej
Gallblåsa	◯ Ja<br>◯ Nej
Annat (t.ex. njure, blindtarm, livmoder eller reproduktionsorgan)	◯ Ja<br>◯ Nej
Har en läkare någon gång sagt att du har haft peritonit som varade i 6 månader eller längre?<br>(Peritonit är en inflammation i bukhinnan.)	◯ Ja<br>◯ Nej

Muskler och skelett<br><br>**Har en läkare någon gång sagt att du har något av följande?**	
Osteoporos, det vill säga benskörhet, som lett till en fraktur?	◯ Ja<br>◯ Nej
Avaskulär nekros?<br>(När en del av benet dör.)	◯ Ja<br>◯ Nej
Osteomyelit?<br>(En infektion i ett ben.)	◯ Ja<br>◯ Nej

Hud<br><br>**Har en läkare någon gång sagt att du har följande?**	
Ett hudsår, alltså ett öppet sår på huden,<br>som varade i 6 månader eller längre?<br>(Detta är inte ett munsår eller ”förkylningsblåsor”.)	◯ Ja<br>◯ Nej

Diabetes	
Har en läkare någon gång sagt att du har diabetes?	◯ Ja<br>◯ Nej

Cancer	
Har en läkare någon gång sagt att du har cancer?	◯ Ja<br>◯ Nej
Vilken typ av cancer? (Lista alla)<br>1.	________________________________
Vilken typ av cancer? (Lista alla)<br>2.	________________________________
Vilken typ av cancer? (Lista alla)<br>3.	________________________________

Menopaus<br><br>**Följande frågor riktar sig endast till kvinnor**	
Denna fråga är endast relevant om du är 40 år eller äldre:<br>Slutade din mens innan du blev 40?	◯ Ja<br>◯ Nej
Berodde detta på att din livmoder opererades bort?	◯ Ja<br>◯ Nej
Denna fråga är endast relevant om du yngre än 40 år:<br>Får du fortfarande din mens?	◯ Ja<br>◯ Nej

Är du gravid eller ammar du?	◯ Ja<br>◯ Nej

Supplemental Material S5. Medication Adherence Self-Report Inventory (MASRI) in Swedish.

Självrapportering om läkemedelsföljsamhet.

Vi vill gärna veta hur mycket av din medicin som du har tagit den senaste tiden. Vi förstår att många personer som tar denna medicin har svårt att ta den regelbundet och ofta missar doser, så vi kommer inte att bli förvånade om även du har missat många doser. Vi behöver veta hur många doser du har missat.

Hur många doser av denna medicin **missade du igår**? (bocka i en ruta)

- ❑ 0
- ❑ 1
- ❑ 2
- ❑ 3
- ❑ Vet inte

Hur många doser av denna medicin **missade du i förrgår**?
(bocka i en ruta)

- ❑ 0
- ❑ 1
- ❑ 2
- ❑ 3
- ❑ Vet inte

Hur många doser av denna medicin **missade du dagen före det**? (för 3 dagar sedan) (bocka i en ruta)

- ❑ 0
- ❑ 1
- ❑ 2
- ❑ 3
- ❑ Vet inte

Hur många doser av denna medicin har du **missat under de senaste 2 veckorna?**
(bocka i en ruta)

- ❑ 0
- ❑ 1
- ❑ 2
- ❑ 3–5
- ❑ 6–10
- ❑ 11–20
- ❑ 20–40
- ❑ Fler än 40
- ❑ Alla
- ❑ Vet inte

När missade du **senast en dos** av denna medicin? (bocka i en ruta)

- ❑ Idag
- ❑ Igår
- ❑ Tidigare i veckan
- ❑ Förra veckan
- ❑ För mindre än en månad sedan
- ❑ För mer än en månad sedan
- ❑ Aldrig
- ❑ Vet inte

Sätt ett **kryss på raden nedan** vid den punkt som **bäst motsvarar din bedömning** om **hur mycket av denna medicin du har tagit under den senaste månaden**.

T.ex. **0 %** betyder att du **inte har tagit någon** medicin
50 % betyder att du har **tagit hälften** av din medicin
100 % betyder att du har **tagit varje enskild dos** av din medicin

0 % 10 % 20 % 30 % 40 % 50 % 60 % 70 % 80 % 90 % 100 %

|_____|_____|_____|_____|_____|_____|_____|_____|_____|_____|

Supplemental Material S6. Compliance Questionnaire of Rheumatology (CQR) in Swedish.

På följande sidor finns ett antal påståenden som patienter med reumatisk sjukdom har gjort. Ange för varje påstående i vilken utsträckning du håller med genom att ringa in den siffra som bäst motsvarar din åsikt.

På följande sidor finns ett antal påståenden som patienter med reumatisk sjukdom har gjort. Ange för varje påstående i vilken utsträckning du håller med genom att ringa in den siffra som bäst motsvarar din åsikt.					
1	Om reumatologen säger åt mig att ta medicinerna så gör jag det.	1<br>Håller inte alls med	2	3	4<br>Håller helt med
2	Jag tar mina antireumatiska mediciner eftersom de gör att jag får mindre problem.	1<br>Håller inte alls med	2	3	4<br>Håller helt med
3	Jag skulle aldrig våga hoppa över mina antireumatiska mediciner.	1<br>Håller inte alls med	2	3	4<br>Håller helt med
4	Om jag kan hjälpa mig själv med alternativa behandlingar föredrar jag det framför vad min reumatolog förskriver.	1<br>Håller inte alls med	2	3	4<br>Håller helt med
5	Jag förvarar alltid mina mediciner på samma plats, därför glömmer jag dem inte.	1<br>Håller inte alls med	2	3	4<br>Håller helt med
6	Jag tar mina mediciner eftersom jag litar fullt ut på min reumatolog.	1<br>Håller inte alls med	2	3	4<br>Håller helt med
7	Det viktigaste skälet till att ta mina antireumatiska mediciner är att jag då kan fortsätta att göra det jag vill.	1<br>Håller inte alls med	2	3	4<br>Håller helt med
8	Jag tycker inte om att ta mediciner. Om jag kan klara mig utan dem så gör jag det.	1<br>Håller inte alls med	2	3	4<br>Håller helt med
9	När jag är på semester händer det ibland att jag inte tar mina mediciner.	1<br>Håller inte alls med	2	3	4<br>Håller helt med
10	Jag tar mina antireumatiska mediciner, för annars behöver man inte gå till reumatologen.	1<br>Håller inte alls med	2	3	4<br>Håller helt med
11	Jag förväntar mig inga mirakel av mina antireumatiska mediciner.	1<br>Håller inte alls med	2	3	4<br>Håller helt med

12	Om man inte står ut med medicinerna kanske man säger: ”släng dem, oavsett vad”.	1 Håller inte alls med	2	3	4 Håller helt med
13	Om jag inte tar mina antireumatiska mediciner regelbundet kommer inflammationen tillbaka.	1 Håller inte alls med	2	3	4 Håller helt med
14	Om jag inte tar mina antireumatiska mediciner varnar kroppen mig.	1 Håller inte alls med	2	3	4 Håller helt med
15	Min hälsa är viktigast av allt och om jag måste ta mediciner för att klara mig så gör jag det.	1 Håller inte alls med	2	3	4 Håller helt med
16	Jag använder en dosett för mina mediciner.	1 Håller inte alls med	2	3	4 Håller helt med
17	Jag gör som läkaren säger.	1 Håller inte alls med	2	3	4 Håller helt med
18	Om jag inte tar mina antireumatiska mediciner får jag mer besvär.	1 Håller inte alls med	2	3	4 Håller helt med
19	Då och då åker jag bort över helgen och tar inte mina mediciner.	1 Håller inte alls med	2	3	4 Håller helt med

Supplemental Material S7. Beliefs About Medicines (BMQ) in Swedish.

Parts of the BMQ were translated to Swedish due to the rest being published in a paper stated in the article.

Dina åsikter om mediciner utskrivna till dig

Vi vill höra vad du tycker om mediciner som du har fått utskrivna. Dessa är påståenden som andra människor har gjort om sina mediciner. Ange hur mycket du håller med om påståendena eller inte genom att bocka i motsvarande ruta.
Inga svar är rätt eller fel. Vi är intresserade av dina personliga åsikter.

- Dessa mediciner ger mig oönskade biverkningar

Dina åsikter om mediciner i allmänhet

Dessa är påståenden som andra människor har gjort om mediciner i allmänhet. Ange hur mycket du håller med om påståendena eller inte genom att bocka i motsvarande ruta.

- Mediciner hjälper många människor att leva ett bättre liv
- I de flesta fall överväger fördelarna med mediciner riskerna
- I framtiden kommer man att utveckla mediciner som kan bota de flesta sjukdomar
- De flesta mediciner är gift
- Mediciner hjälper många människor att leva längre

Supplemental Material S8. Method of translation from English to Swedish.

First, an experienced medical translator with Swedish as mother tongue translated the questionnaires from English to Swedish. Then, an experienced medical translator with English as mother tongue translated from Swedish into English. The translator did not have access to the English original to ensure it did not influence the translation. Any discrepancies between the original English and back translated English were examined by the Swedish translator. All ambiguities in the translation were identified and clarified, and the Swedish translation was adjusted accordingly.

Supplemental Material S9. Custom survey questions in English and in Swedish.

**Patient information**	
Date of birth	________________ (YYYYMMDD)
Four last digits in civic registration number	________________ (XXXX)
E-mail	________________
Sex	◯ Female<br>◯ Male
Living situation	◯ Alone<br>◯ Not alone
Postal code	________________
Are you currently employed or running your own business?<br>(If you answered "Yes", go to question 1)(If you answered "No", go to question 2)	◯ Yes<br>◯ No
1) Specify if it is a full-time or part-time job	◯ Full-time (40 hours per week, or more)<br>◯ Part-time (less than 40 hours per week)
2) Specify what your occupation is	◯ Student<br>◯ Retired<br>◯ Other
Highest education level achieved	◯ University<br>◯ High school<br>◯ Less than a high school diploma
Height	________________ (cm)
Weight	________________ (kg)
Smoking status<br>(If you answered "Never" go to Disease-specific and treatment-related questions)<br>(If you answered "Current" go to questions 1-2)<br>(If you answered "Former" go to questions 3-5)	◯ Never<br>◯ Current<br>◯ Former
1) On average how many cigarettes do you smoke per day?	________________
2) For how many years have you been smoking?	________________

3) When did you quit smoking?	____________ (YYYY)
4) On average how many cigarettes did you use to smoke per day?	____________
5) For how many years did you smoke?	____________

Disease-specific and treatment-related questions

Ongoing medication(s)<br>(If you answered "Prednisolon", go to question 1.<br>(If you answered "Cortisone other than Prednisolon", go to questions 2-3)<br>(If you answered "Other medications", go to question 4)	☐ Prednisolon<br>☐ Cortisone other than Prednisolon<br>☐ Plaquenil<br>☐ Klorokinfosfat<br>☐ Azatioprin/Imurel<br>☐ Mykofenolatmofetil/Cellcept<br>☐ Methotrexate (pills)<br>☐ Metoject (injection)<br>☐ Sandimmun<br>☐ Benlysta (infusion)<br>☐ Benlysta (injection)<br>☐ Mabthera<br>☐ Other medication(s)<br>(More than one option is possible)
1) Prednisolon dose (mg/kg)	____________
2) Specify which cortisone other than Prednisolon	____________
3) Cortisone (other than prednisolon) dose (mg/kg)	____________
4) Specify what other medication(s)	____________
When you sum up all the medication(s) you take for one typical day, how many separate pills and/or injections do you take in total?	◯ 0<br>◯ 1-3<br>◯ 4-5<br>◯ >5

What other diseases have you been diagnosed with, if any?<br><br>(If you answered "Kidney disease", go to question 1-2)<br>(If you answered "Other diagnoses", go to question 3)	☐ None<br>☐ Fibromyalgia<br>☐ Depression<br>☐ High blood pressure<br>☐ High blood cholesterol<br>☐ Diabetes<br>☐ Kidney disease<br>☐ Osteopenia/osteoporosis<br>☐ Osteoarthritis<br>☐ Rheumatoid arthritis Sjögren's<br>☐ syndrome Antiphospholipid<br>☐ syndrome Other diagnoses<br>☐<br>(Fill in all that apply)
1) Is your kidney disease due to SLE?	○ Yes<br>○ No
2) What is the reason for your kidney disease if not SLE?	________________________
3) Specify which diagnoses	________________________
**Reflections on medications**	
Click the boxes that best describe reasons for difficulties taking your medication as prescribed<br><br>(If you answered "I am worried that I will experience side effects of my medication", go to question 1)<br>(If you answered "Other", go to question 2)	☐ None-I don't have difficulties taking my medication as prescribed.<br>☐ I am unhappy with my physician or healthcare.<br>☐ I am afraid that my medication increases the risk to get a Covid-19 infection.<br>☐ I am worried that my medication increases the risk to get a more severe Covid-19 infection.<br>☐ I am worried that I will experience side effects of my medication.<br>☐ When I don't feel in a good shape I might not take my medications on a specific day.<br>☐ Too many different medications to keep track on.<br>☐ Too many different timeslots to keep track on.<br>☐ I believe my symptoms will not be improved.<br>☐ My disease is not active at the moment.<br>☐ It takes too long for my symptoms to be improved.<br>☐ My financial issues prevent me from collecting my prescription from the pharmacy.<br>☐ Other:<br>(More than one option is possible)
1) Which side effects are you mostly worried about?Please state the medications that apply and specific side effects for each one of those	________________________
2) Specify what reasons	________________________
What do you think would help you take your medications as prescribed?	________________________

COVID-19	
Have you discontinued any of your medications because of the Covid-19 outbreak?<br>(If you answered "Yes", go to question 1)	◯ Yes<br>◯ No

1) Specify which medications and why

____

Interview	
Would you be willing to be interviewed over the phone or in person in order for us to get a deeper understanding of your answers? (If you answered "Yes", go to question 1)	◯ Yes<br>◯ No

1) Please, give us your contact information:

____

Factors associated with non-adherence to medications in systemic lupus erythematosus: results from a Swedish survey

Patientinformation	
Födelsedatum	______________ (ÅÅÅÅMMDD)
Fyra sista siffrorna i personnumret	______________ (XXXX)
E-mail	______________
Kön	◯ Kvinna<br>◯ Man
Hushåll	◯ Singel<br>◯ Ej singel
Postnummer	______________
Är du anställd /egenföretagare?<br>(Om "Ja", gå till fråga 1)<br>(Om"Nej", gå till fråga 2)	◯ Ja<br>◯ Nej
1) Är det heltid eller deltid?	◯ Heltid (40 timmar i veckan, eller mer)<br>◯ Deltid (mindre än 40 timmar i veckan)
2) Vad är din sysselsättning?	◯ Student<br>◯ Pensionär<br>◯ Annat
Utbildningsnivå	◯ Högstadiet<br>◯ Gymnasiet<br>◯ Universitet
Längd	______________ (cm)
Vikt	______________ (kg)
Röker du?<br>(Om "Aldrig", gå till sjukdoms-/läkemedelsrelaterade frågor)<br>(Om" Ja", gå till fråga 1-2)<br>(Om " Har rökt tidigare men slutat", gå till fråga 3-5)	◯ Aldrig<br>◯ Ja<br>◯ Har rökt tidigare men slutat
1) Hur många cigaretter i genomsnitt brukar du röka per dag?	______________
2) I hur många år har du rökt?	______________

3) När slutade du röka?

____
(ÅÅÅÅ)

4) Hur många cigaretter i genomsnitt brukade du röka per dag?

____

5) Under hur många år rökte du?

____

Sjukdoms-/läkemedelsrelaterade frågor

Nuvarande läkemedel

(Om du fyllt i "Prednisolon", svara på fråga 1)
(Om du fyllt i "Annan kortison än Prednisolon", svara på fråga 2-3)
(Om du fyllt i "Annat läkemedel", svara på fråga 4)

- ☐ Prednisolon
- ☐ Annan kortison än Prednisolon
- ☐ Plaquenil
- ☐ Klorokinfosfat
- ☐ Azatioprin/Imurel
- ☐ Mykofenolatmofetil/Cellcept
- ☐ Methotrexate (tabletter)
- ☐ Metoject (injektion)
- ☐ Sandimmun
- ☐ Benlysta (infusion)
- ☐ Benlysta (injektion)
- ☐ Mabthera
- ☐ Annat läkemedel

(Mer än ett alternativ är möjligt)

1) Prednisolon dos (mg/kg)

____

2) Ange vilket kortison (annan än Prednisolon)

____

3) Kortisondos (annan än Prednisolon) (mg/kg)

____

4) Ange vilket läkemedel

____

Totalt sett på en dag, när du summerar all medicin du tar, hur många olika tabletter och/eller injektioner blir det då?

- ◯ 0
- ◯ 1-3
- ◯ 4-5
- ◯ >5

**Ange annan diagnos utöver SLE**<br><br>(Om du fyllt i"Njursjukdom", svara på fråga 1-2)<br>(Om du fyllt i "Andra diagnoser", svara på fråga 3)	☐ Ingen<br>☐ Fibromyalgi<br>☐ Depression<br>☐ Högt blodtryck<br>☐ Höga kolesterolvärden<br>☐ Diabetes<br>☐ Njursjukdom<br>☐ Benskörhet<br>☐ Artros<br>☐ Ledgångsreumatism<br>☐ Sjögrens syndrom<br>☐ Antifosfolipid syndrom<br>☐ Andra diagnoser<br>(Mer än ett alternativ är möjligt)
1) **Beror din njursjukdom på SLE?**	◯ Ja<br>◯ Nej
2) **Vad är orsaken till din njursjukdom om inte SLE?**	____________________
3) **Ange vilka diagnoser**	____________________

Reflektioner kring dina läkemedel

**Fyll i de påståenden som du tycker stämmer med varför det har varit svårt att ta läkemedel enligt ordination**<br><br>(Om du svarat "Jag är orolig över biverkningar av mina läkemedel", svara på fråga 1)<br>(Om du svarat "Annat", svara på fråga 2)	☐ Jag tar mina läkemedel som de ordinerats<br>☐ Jag är missnöjd med min läkare eller sjukvård<br>☐ Jag är rädd att mina läkemedel ska göra mig mer mottaglig till en Covid-19-infektion<br>☐ Jag är rädd att mina läkemedel ska göra mig mer mottaglig till en mer allvarlig Covid-19-infektion<br><br>☐ Jag är orolig över biverkningar av mina läkemedel<br><br>☐ När jag känner mig dålig en viss dag<br>☐ För många olika läkemedel att hålla reda på<br>☐ För många olika tidpunkter att hålla reda på då läkemedlen ska tas<br>☐ Jag tror inte att mina symtom kommer förbättras av läkemedlen<br>☐ Saknar motivation att fortsätta ta mina läkemedel eftersom jag mår bättre<br>☐ Det tar för lång tid innan mina sjukdomssymtom förbättras<br>☐ Jag har inte råd att hämta ut mina recept från apoteket<br>☐ Annat<br>(Mer än ett alternativ är möjligt)
1) **Ange vilka biverkningar samt vilka läkemedel?**	____________________
2) **Ange andra anledningar**	____________________
**Vad tror du skulle kunna få dig att ta dina läkemedel precis som ordinerat?**	____________________

COVID-19

Har du slutat ta något av dina läkemedel på grund av rådande Covid-19 pandemi? (Om" Ja", gå till fråga 1)

◯ Ja
◯ Nej

1) **Ange vilka läkemedel och anledning**

____

Intervju

Kan du tänka dig att medverka i en intervju per telefon eller personligen kring dessa frågeställningar där jag får möjlighet att förstå dina tankar lite mer?

(Om" Ja", gå till fråga 1)

◯ Ja
◯ Nej

1) **Vänligen ange din kontaktinformation:**

____

References

1. Cramer JA, Mattson RH, Prevey ML, Scheyer RD, Ouellette VL. How often is medication taken as prescribed? A novel assessment technique. Jama. 1989;261(22):3273-7.
2. Du X, Chen H, Zhuang Y, Zhao Q, Shen B. Medication Adherence in Chinese Patients With Systemic Lupus Erythematosus. J. 2020;26(3):94-8.
3. Zhang L, Lu GH, Ye S, Wu B, Shen Y, Li T. Treatment adherence and disease burden of individuals with rheumatic diseases admitted as outpatients to a large rheumatology center in Shanghai, China. Patient preference and adherence. 2017;11:1591-601.
4. Garcia-Gonzalez A, Richardson M, Garcia Popa-Lisseanu M, Cox V, Kallen MA, Janssen N, et al. Treatment adherence in patients with rheumatoid arthritis and systemic lupus erythematosus. Clinical rheumatology. 2008;27(7):883-9.
5. Zhang L, Luan W, Geng S, Ye S, Wang X, Qian L, et al. Lack of patient education is risk factor of disease flare in patients with systemic lupus erythematosus in China. BMC Health Serv Res. 2019;19(1):378.
6. Mendoza-Pinto C, Garcia-Carrasco M, Campos-Rivera S, Munguia-Realpozo P, Etchegaray-Morales I, Ayon-Aguilar J, et al. Medication adherence is influenced by resilience in patients with systemic lupus erythematosus. Lupus. 2021:9612033211004722.
7. Karlson EW, Daltroy LH, Rivest C, Ramsey-Goldman R, Wright EA, Partridge AJ, et al. Validation of a Systemic Lupus Activity Questionnaire (SLAQ) for population studies. Lupus. 2003;12(4):280-6.
8. Pettersson S, Svenungsson E, Gustafsson J, Moller S, Gunnarsson I, Welin Henriksson E. A comparison of patients' and physicians' assessments of disease activity using the Swedish version of the Systemic Lupus Activity Questionnaire. Scand J Rheumatol. 2017;46(6):474-83.
